# Brief interventions 2.0: a new agenda for alcohol policy, practice and research

**DOI:** 10.1186/s12992-024-01031-1

**Published:** 2024-04-19

**Authors:** Duncan Stewart, Mary Madden, Jim McCambridge

**Affiliations:** 1 School of Social Sciences and Professions, London Metropolitan University, London, N7 8DB UK; 2 Department of Health Sciences, University of York, York, YO10 5DD UK

**Keywords:** Alcohol, Global health, Prevention, Health systems, Alcohol policy, Brief interventions

## Abstract

**Background:**

Alcohol problems are increasing across the world and becoming more complex. Limitations to international evidence and practice mean that the screening and brief intervention paradigm forged in the 1980s is no longer fit for the purpose of informing how conversations about alcohol should take place in healthcare and other services. A new paradigm for brief interventions has been called for.

**Brief interventions 2.0:**

We must start with a re-appraisal of the roles of alcohol in society now and the damage it does to individual and population health. Industry marketing and older unresolved ideas about alcohol continue to impede honest and thoughtful conversations and perpetuate stigma, stereotypes, and outright fictions. This makes it harder to think about and talk about how alcohol affects health, well-being, and other aspects of life, and how we as a society should respond. To progress, brief interventions should not be restricted only to the self-regulation of one’s own drinking. Content can be orientated to the properties of the drug itself and the overlooked problems it causes, the policy issues and the politics of a powerful globalised industry. This entails challenging and reframing stigmatising notions of alcohol problems, and incorporating wider alcohol policy measures and issues that are relevant to how people think about their own and others' drinking. We draw on recent empirical work to examine the implications of this agenda for practitioners and for changing the public conversation on alcohol.

**Conclusion:**

Against a backdrop of continued financial pressures on health service delivery, this analysis provokes debate and invites new thinking on alcohol. We suggest that the case for advancing brief interventions version 2.0 is both compelling and urgent.

## Background

In an era of restrictions on health budgets and ageing populations, alcohol problems are increasing across the world [1], generating new treatment demand and need for interventions. This is particularly so in low- and middle-income countries (LMICs) where alcohol markets are expanding and harm per litre consumed is greatest [2], whilst within high income countries, alcohol makes health inequalities worse [3]. Substantial mental health comorbidities are increasingly the norm in treatment systems [4], and physical health comorbidities are becoming more visible in older populations [5].

The obvious response to this situation is to make a better case to win more resources, resist cuts and defend what exists. We suggest, however, that this is not enough, and that new thinking is now needed. Health systems struggle to embrace prevention across the board [6]. “Brief interventions” originated in the public health understanding of alcohol. The nature of the challenge has changed in fundamental ways in recent decades, and their limitations are better understood. This makes timely a re-appraisal, reconnecting to contemporary public health ideas and evidence.

We propose that we should now reimagine the contents and aims of brief interventions, and how they might act in synergy with other efforts to address the avoidable damage done by alcohol.

### The brief intervention concept

A little under half a century ago, the rise of the new public health movement made health promotion and disease prevention central to improving population health. Alcohol was highly relevant to this development. The World Health Organisation brought together alcohol researchers in a major programme that developed the AUDIT screening tool [7] and undertook a randomised trial that demonstrated that it was possible to have conversations with people in primary care that led them to reduce drinking [8]. This represented a new way of responding to alcohol problems; avoiding waiting until treatment for well-established problems was sought.

Many of the key research questions identified in a “golden age” of research advances in the late 1980s and early 1990s remain unanswered today [9]. There were theoretical weaknesses in the advice and counselling interventions developed and practitioners did not implement them in routine practice [10]. Much of the available evidence is from high-income countries, with relatively few trials conducted in LMICs [11]. Conflicting findings and the limitations of the large body of international literature have received too little attention [12]. It is perhaps most appropriately interpreted as demonstrating efficacy; recent large trials in naturalistic conditions demonstrate that confident claims of effectiveness are misplaced [12]. As a result, programmes may attain reach, which is itself challenging, but cannot be expected alone to deliver health impacts in populations where they are implemented [13]. The digital alcohol intervention literature has evolved in similar ways, with much promise in early studies, but with near exclusive reliance on self-reported outcomes not routinely included within risk of bias assessments, large trials with different findings than smaller trials, and substantial unexplained heterogeneity in meta-analyses [10].

Over the last 10 years a consensus has taken hold in the field that a change in direction is needed; a chronic disease paradigm is one possibility [14, 15], and more extensive development of digital interventions another [16]. Our thinking centres on the unhelpful dislocation of brief interventions from wider alcohol policy measures everywhere. We note the very different contexts for the audience for this paper. These include readers in LMICs where there are no brief intervention programmes or alcohol policy measures. And also, readers in high income countries where such programmes provide important care services (such as screening, brief intervention and referral to treatment (SBIRT) in the U.S.) with or without otherwise well-developed alcohol policies.

### The alcohol challenge for health systems

Adults, and children, are exposed to alcohol marketing in competition with relatively impotent health promotion messages [17]. Norms are shaped early in life, and drinking and heavy drinking is normalized in many countries. With the aid of new technologies, marketing is getting ever more sophisticated [18]. The environment is also one in which the persistence of stereotypical ideas of the so called ‘alcoholic’ and stigmatized images of alcohol problems obstruct broader thinking about the nature and impacts of alcohol harms [19]. Public understanding has not been informed by the developing science: there remains no consensus in the research community on what is an alcohol problem [20].

Locating an alcohol problem within the individual, consonant with neoliberal ideas that people are responsible for everything in their own lives, invisibilises government and business roles and responsibilities in causing alcohol problems [21]. Large corporations typically make a potentially dangerous drug widely available, encourage people to use it, shape government policy to place few restrictions on its use, and then blame those who end up having problems with it [22]. The ethical issues here warrant attention, especially as problems for drinkers cause families and communities to have alcohol problems too [23].

The structure of the alcohol industry increasingly resembles tobacco, especially in beer and spirits [24]. The largest companies are now highly profitable and operate globally, whereas only 30 years ago they were national operators. They are connected to tobacco companies in various ways [22, 25] and use the same approaches; selling themselves as part of the solution not part of the problem, with the resources needed to do that effectively [26]. Alcohol policy interference is unrestricted, whereas tobacco has been curbed [27].

The power of alcohol industry marketing needs to be restricted if we are to help people to manage their alcohol consumption in ways which limit damage to health and well-being. Brief interventions have sought to help people avoid or manage problems with alcohol, but that is harder to do now in the contexts of lifetime exposure to industry and other social influences, deepening inequalities and weakened capacity or willingness to manage unhealthy commodity industries [28]. It is perhaps unsurprising that the original ambitions for brief interventions have yet to be realised convincingly when prices are low, availability easy and norms encourage more drinking [29]. To progress, we need to recognise that, for many reasons, alcohol and the problems it causes may be challenging to identify and discuss with individuals. Invidiously, this is especially so when drinking is heavier. We need to find new ways of talking about all of this.

### Ways forward for brief interventions 2.0

Brief interventions are simply conversations about alcohol, so how might brief interventions 2.0 (BI 2.0) make them more powerful?

Firstly, we should not continue to think of brief interventions as only to do with self-regulation of one’s own consumption, in isolation from personal health and social contextual factors. This means re-orientating brief interventions to the damage done, directly and indirectly, by a toxic and carcinogenic drug and the enormous burden it places on health services and society. There is no entirely safe dose [30, 31] and people with existing health problems are particularly vulnerable to additional harms from interference with the effects of medications designed to benefit health, including on adherence [5]. These impacts should be integral to routine discussions about treatments, conditions and wider well-being, rather than the current practice of regarding alcohol as a separate, “lifestyle” issue. Such constructs inhibit patients and practitioners in approaching alcohol and its harms meaningfully.

Brief intervention content has also failed to keep pace with and take account of contemporary evidence on the wider determinants of health [32–34], the continued challenges they present for policy and practice [35], and the particular vulnerability of the most disadvantaged to alcohol harms [36]. Stigmatising attitudes, cultural norms, price, availability, and industry marketing are important influences on drinking behaviour [37], so we need brief interventions to address these issues too. Having a wider content repertoire may help people to think differently about the place of alcohol in their lives, and in wider society. This may be particularly apposite where there is media attention or concurrent policy debates and developments; brief intervention programmes could be designed to incorporate attention to them. In the absence of policy innovations, in all countries where alcohol consumption is widespread, there is mass media content on alcohol; alcohol harm hides in plain sight. Such influences should not only be more fully recognised as the context in which conversations about one’s own drinking takes place but can also be a part of that conversation. We should be talking about whatever is interesting about alcohol to the people we have the time and opportunity to talk with.

A further proposition follows on from this. Where new policy measures are being considered, adopted, or implemented, or where there are public health campaigns, brief intervention programmes could form a key part of more integrated comprehensive alcohol strategies. Innovative resources, in diverse media, can be produced that support conversations taking place that reinforce the effects of other interventions. Such materials may be designed to prompt thinking, enhance readiness and willingness to discuss alcohol, with health and other services being able to take further the implications for the needs they serve. Adjusting programme aims in this way may seem obvious, and is very much in line with the original aspirations for brief interventions as instruments of public health improvement, so it is disappointing that possible synergies of this kind have been so little studied. Opportunities for so doing should be grasped when they arise.

Progressing BI 2.0 is contingent on overcoming the prevalent idea that labelling people as ‘alcoholics’ or ‘problem drinkers’ provides the most helpful way of thinking about this subject [38]. It does not. In fact, it gets in the way [39]. People can have many problems, and the more one drinks the more likely it is that alcohol will complicate things, often in ways that are difficult to appreciate [40]. Perhaps, focusing on what may seem the less serious initially may help problem recognition, such as having a hangover, missing a day’s work, or an “accident”. There is something to consider in these examples that it might be helpful to discuss rather than disregard.

At the population-level, it is for all of us and our policy makers to consider how far and in which ways we have an alcohol problem [41]. This does not mean denying that alcohol also brings pleasure and other benefits. Decision-making around use of this drug needs to be more rational, because currently it is too pressured by pro-consumption influence and relics of past ways of thinking. Ultimately, development of BI 2.0 requires a candid public conversation about how alcohol and alcohol problems interfere with the lives that people want to live.

### Putting BI 2.0 ideas into practice

In busy and over-burdened health services, it may at first seem far-fetched to expect that BI 2.0 will appeal to practitioners or their managers, especially so if presented as a new or additional task. A better approach is to present it as a way of responding to what patients already bring with them. We have been working with clinical pharmacists in primary care to help them briefly explore whether there are any alcohol connections to why patients are presenting or have been asked to attend [42]. To be a conscientious professional, many health care practitioners need to be able to discuss alcohol for medication safety, adherence, and effectiveness reasons, as well as the implications of alcohol for many conditions. Seeing alcohol as a drug makes it not just legitimate but important to raise and integrate clinically into consultations for both professionals and patients [43]. Most importantly for the patient, alcohol is discussed in the context of their health and what matters to them, using their own language and terminology, where the relevance is clear.

If people make connections between alcohol, medicines regimes, other daily activities and their health, then this invites broader social contexts into discussions. Too often, conversations in health and care settings about alcohol are too brief, too crude, heavily moralised, paternalistic and all too easy to ignore, when they are not avoided altogether [43, 44]. Confident, skilled practitioners can offer support that helps people make their own decisions about alcohol use, navigating the cultural influences that make talking about alcohol more challenging than it needs to be. For professionals as well as patients. Much existing information and other tools for discussion look dry and dull, especially in comparison to industry investment in engaging marketing materials. So too our digital resources. We need content that is appealing, lively, and engaging to capture and keep hold of attention. We should design material that people will want to share with others in their social networks. Intimacy also matters; content that resonates personally is to be prized because that is tapping into what’s important to the person.

For all these reasons, and more, these conversations need to be skilfully handled or the deleterious effects of alcogenic cultural baggage will continue to hinder us. That is why we think that working with practitioners and opening up practice development issues is a promising place to move forward with BI 2.0 (Table 1).

**Table 1:** BI 2.0 ideas for practitioners

Alcohol as a drug
○ Alcohol is a health harming drug that is often overlooked and is clinically relevant to all practitioner roles
○ Good practice necessarily requires discussion of alcohol, particularly in medications related work
○ Attention to alcohol can be integrated into routine consultations efficiently, allowing exploration of connections to medicines, conditions, adherence issues, and health more broadly
Avoid moralising in discussing alcohol
○ Invite people to talk about their drinking in their own terms
○ Help people to think through whether drinking affects medicine use, conditions and health, to understand and manage the risks for themselves
○ Ascertain willingness to discuss alcohol in daily lives and the influences that shape drinking patterns
○ Appreciate that alcohol may be very briefly focused on for many, and a lot for some, with life course perspectives relevant
○ Invite people to talk about their drinking in their own terms
Engage with alcohol issues in a person-centred manner
○ Appreciate that alcohol can be a difficult topic to raise in consultations for both parties, and can be raised in ways that avoid negative connotations
○ Do not push discussion of alcohol on to unwilling patients
○ Support patients in thinking further about, and making, decisions to improve their health and well-being by encouraging discussion of their concerns and priorities
○ Provide alternatives to unhelpful ways of thinking in addressing stereotypes, myths and stigma as opportunities arise

## Conclusions

There is global recognition that tackling alcohol harms requires a multifaceted approach, incorporating restrictions on availability, advertising, and pricing policies as well as facilitating access to brief interventions [34, 45]. We have presented ideas for progressing BI 2.0, which orientates intervention content and aims to these other elements and the larger contexts, and puts prevention at the heart of policy and practice. This requires a system-wide approach that avoids the pitfalls of focusing on stereotyped notions of problem drinking, highlights the need to strengthen the wider public conversation on alcohol and promotes synergies with developing alcohol policies. Our intention is to provoke discussion, debate, study and action, and we suggest this must proceed with urgency.

## Data Availability

Not applicable.

## Abbreviations

BI: Brief interventions
LMICs: Low- and middle-income countries
SBIRT: Screening, brief intervention and referral to treatment
